# A Real-Time, Automatic, and Dynamic Scheduling and Control System for PET Patients Based on Wearable Sensors

**DOI:** 10.3390/s21041104

**Published:** 2021-02-05

**Authors:** Shin-Yan Chiou, Kun-Ju Lin, Ya-Xin Dong

**Affiliations:** 1 Department of Electrical Engineering, College of Engineering, Chang Gung University, Kwei-Shan, Tao-Yuan 333, Taiwan; ansel@mail.cgu.edu.tw (S.-Y.C.); cindy_doong@wistron.com (Y.-X.D.); 2 Department of Nuclear Medicine, Linkou Chang Gung Memorial Hospital, Tao-Yuan 333, Taiwan; 3 Department of Medical Imaging and Radiological Sciences, Chang Gung University, Kwei-Shan, Tao-Yuan 333, Taiwan

**Keywords:** PET, automatic scheduling, control system, wearable sensors, Bluetooth Beacon

## Abstract

Positron emission tomography (PET) is one of the commonly used scanning techniques. Medical staff manually calculate the estimated scan time for each PET device. However, the number of PET scanning devices is small, the number of patients is large, and there are many changes including rescanning requirements, which makes it very error-prone, puts pressure on staff, and causes trouble for patients and their families. Although previous studies proposed algorithms for specific inspections, there is currently no research on improving the PET process. This paper proposes a real-time automatic scheduling and control system for PET patients with wearable sensors. The system can automatically schedule, estimate and instantly update the time of various tasks, and automatically allocate beds and announce schedule information in real time. We implemented this system, collected time data of 200 actual patients, and put these data into the implementation program for simulation and comparison. The average time difference between manual and automatic scheduling was 7.32 min, and it could reduce the average examination time of 82% of patients by 6.14 ± 4.61 min. This convinces us the system is correct and can improve time efficiency, while avoiding human error and staff pressure, and avoiding trouble for patients and their families.

## 1. Introduction

Hospital medical equipment resources are often in short supply. Equipment scheduling control is an important issue. This issue is roughly divided into three categories: “reservation scheduling”, “real-time scheduling” and “smart device applications”.

### 1.1. Reservation Scheduling

There are many different outpatient clinics in the hospital, as well as multiple physical examination devices. However, the patient examination needs exceed the medical resources. The resources provided by the hospital are in short supply; thus, good scheduling is required to resolve the problem. Burdett et al. [1] robustly scheduled appointments by strategically inserting buffer times, and they combined simulated annealing and evolutionary search to improve schedule ability. Jiang et al. [2] found that most studies on appointment systems assume that patients arrive at the appointment time, but the actual situation is not the case. Therefore, they proposed a random planning appointment scheduling system considering late patients, using Benders decomposition combined with the sample average approximation (BD-SAA) technique to find the best appointment time that reaches the least patient waiting time and doctor idle time. Qiu et al. [3] proposed algorithms using NSGA-II (non-dominated sorting genetic algorithm II) and MOEA/D (multiobjective evolutionary algorithm based on decomposition) for a single MRI device to arrange the order of appointment and appointment time for patients of different types of examination. Wu et al. [4] calculated the length of MRI examinations in different body parts and assigned corresponding MRI equipment, and then combined the statistical data to determine the length of each examination to increase the usage of MRI equipment and reduce the patient waiting time. Xiao et al. [5] proposed a method based on genetic algorithms for the nuclear medicine department to solve complex scheduling problems with many conditions. The background of [5] is similar to this article, but their method can only be applied in appointment situations, while our proposed method can estimate the patient waiting time for various tasks in real time.

However, appointment scheduling can only maximize the number of patients by arranging the examination dates and order for patients, whereas it cannot solve the problem that patients do not arrive at the hospital according to the appointment time. In fact, on the examination day, patients often arrive early or late, do not arrive, or join examination temporarily. This will widen the gap between the actual and the appointment time, and the patients’ original examination time may change due to these factors. In order for patients to understand the on-site conditions, this paper proposes a real-time automatic scheduling and control system. It is an effective method to provide on-site scheduling and to estimate inspection time, and it can optimize the entire inspection process and ease the workload of medical staff.

### 1.2. Real-Time Scheduling

Many scholars have proposed solutions to the random variability of on-site patients. Azadeh et al. [6] proposed a mixed-integer linear programming model in order to solve the scheduling difficulties caused by multiple examinations in a single patient, and they combined genetic algorithms to solve the problem of finding the best solution. Peng et al. [7] proposed a method using discrete event simulation combined with genetic algorithms to maximize the number of patients served. This method can dynamically schedule patients who were previously booked, were booked on-site, or waited on-site. However, they [6,7] can only provide queue status (number of patients, queue position), whereas they cannot provide patients with estimated examination time.

Many scholars [8,9,10] proposed methods for outpatients to arrive at the scene close to the consultation time. Montecinos et al. [8] used the method of particle filters to estimate the waiting time of consultation using historical data of patient visit time and new input data. Tantitharanukul et al. [9] developed an estimated waiting time system (WTE) based on the queuing theory, and they used the MQTT (Message Queue Telemetry Transport) protocol to transfer the waiting time and number to the patient’s cell phone. Obulor et al. [10] also used the queuing theory to develop an appointment queuing system to provide better resource utilization to solve the problem of long waiting times for patients. These studies [7,8,9,10] estimated the time for a single treatment, while the system proposed in this paper is for multistage treatments such as positron emission tomography (PET), and it can dynamically estimate the start and end time of each different stage, as well as instantly announce this time information on the public screen or send it to the patient’s mobile phone.

Because appointed patients are sometimes late or absent, which affects the time of patients who are registered and waiting at the scene, the original schedule must be constantly modified and updated in real time. The above studies [9] and [11] aimed at improving the situation. Qu et al. [11] used the Markov model to optimize the scheduling of patients waiting on-site, and they proposed a heuristic algorithm for other possible situations. The model divides the day’s visits into multiple time slots and then queues the patients into each time slot one by one.

Ruth Luscombe et al. [12,13] developed job shop scheduling, combined with heuristic algorithms, to quickly coordinate the activities and resources of emergency departments. Wiesche et al. [14] proposed an optimization model of optimal reservation of capacity for appointments (ORCA), a system that can arrange patients without appointments and patients who already have appointments, as well as provide them on days when the demand for patients is low. This allows maximizing capacity for appointments to potentially treat a walk-in patient on a high-demand day. The system of Kortbeek et al. [15] used the day process model and access process of scheduled arrivals model to balance the “waiting machine time for patients without appointments” and “examination time for patients with appointments”, and they divided the time of day into multiple time slots to arrange patients.

However, in a PET examination, it is necessary to consider that each patient has a different medicine cycle time at different examination sites, and there are also cases where the examination fails and the examination is repeated. Therefore, these studies cannot solve the problem of real-time scheduling for PET inspections. The method we propose takes into account the drug circulation time and solves the problem of inspection failure through dynamic rearrangement.

### 1.3. Smart Device Applications

Some patients have multiple examinations in the hospital, and some examinations have multiple tasks [16,17,18]. Without proper time control, patients will spend a lot of time waiting. By recording the start and end time of each task, we can analyze the data and reduce patient waiting time.

Frisby et al. [19] proposed a new method for controlling patients in the emergency room. Each doctor wore a medical Bluetooth device and installed a Bluetooth signal receiver in each bed. Within the acceptable range, the treatment time was automatically recorded, which effectively reduced the doctor’s work. Ewing et al. [20] and Elnahrawy et al. [21] used an instant positioning system to collect data on the patient’s movements in the hospital and the time spent at each station to analyze the data and improve the hospital workflow. Kortbeek et al. [15] developed a frame that combines data collection with electronic medical records for data analysis. This method effectively understands the flow of patients in various areas of the hospital and can be used to control different situations in the future. Naruse et al. [22] set up a Beacon signal receiver made using Raspberry Pi in the room to detect the Beacon signal. When someone enters the room, they identify the Beacon, transmit the Beacon data to the database, and calculate the duration of the Beacon carrier stay.

This paper proposes a method for arranging beacon signal receivers (i.e., mobile phones) in various areas of PET, receiving signals from patients wearing Beacon devices, and automatically recording the time of patients during various inspection tasks, saving medical staff time for manual transcription and improving overall automation.

### 1.4. Current Status and Issues of PET Patient Scheduling

Many PET standard tasks are performed during the inspection, and the cycle time of the medicine varies from part to part. Currently, medical staff use manual methods to calculate the estimated scan time for each PET device. However, the number of PET scanning devices is extremely small, while the number of patients is quite large. Moreover, there are many changing conditions such as different arriving times of both scheduled and temporary patients, constant calculations for different examination items and different beds and drug waiting times, and rescanning requirements for some patients who failed to scan.

Manual scheduling is very error-prone and puts pressure on staff. Coupled with this constantly changing schedule, it is difficult to announce the estimated time to patients and their families in real time, causing family members to constantly ask medical staff about various conditions such as waiting time, which not only consumes the medical staff’s physical and mental strength, but also results in unsmooth processes.

Previous researches [1,2,3,4,5,6,7,8,9,10,11,12,13,14,15,16,17,18,19,20,21,22] proposed algorithms for specific examinations to solve the problem of equipment resource allocation, as well as calculate outpatient visit time for outpatient clinics. However, there is currently no research on improving the PET process. Therefore, we propose an automatic scheduling method to improve the current PET patient scheduling problem.

### 1.5. Overview of Results

This paper proposes a real-time, automatic, and dynamic scheduling and control system for PET patients with wearable sensors (Bluetooth Beacon) positioning. This system can automatically schedule, estimate, and update the start and end time of various tasks of PET patients during the examination, and it can automatically allocate beds and real-time announce schedule information, allowing the schedule being automated, instant, and almost optimized. This can greatly reduce the work of medical staff, avoid human error, improve medication safety, and allow medical staff, patients, and family members to instantly check the progress of the inspection and estimated waiting time, reducing the need for patients and family members to ask medical staff. We also implemented the proposed method in the Android system to prove the feasibility of the system.

We also developed an app (i.e., a mobile phone application) that can automatically collect data. This app collected various “manually scheduled” data from 200 actual patients in the Department of Nuclear Medicine of Linkou Chang Gung Hospital as a control group. We also fed the initial situation of the same 200 original data collected by the above-implemented Android system and obtained various “auto-scheduled” data as the experimental group. It was found that the average time difference between the “manually scheduled control group” and the “auto-scheduled experimental group” was 7.32 min, indicating the correctness of this method. Furthermore, the “auto-scheduled experimental group” could reduce the average examination time of 82% of patients by 6.14 ± 4.61 min, which proves the progress of this method.

### 1.6. Our Contribution

This paper is the first to propose a novel and real-time automatic scheduling and control system for PET patients. The system provides the following functions: (1) real-time automatic scheduling, (2) scheduling for different medicines, (3) dynamic update of prediction time, (4) immediate provision of predictive inspection time, (5) automatic allocation of general and specific beds, (6) automatic detection of all patients’ tasks periods, (7) automatic detection and prediction of examination room conditions, and (8) instant rescheduling. The major contribution is that the proposed system can greatly reduce the work of medical staff, avoid human error, improve medication safety, and allow medical staff, patients, and family members to instantly check the progress of the inspection and estimated waiting time, reducing the need for patients and family members to ask medical staff. The value-added contribution is that the system can reduce the average examination time of 82% of patients by 6.14 min. Moreover, our method has portability, which means that our detailed methods and implementation content can be easily transplanted to PET patient scheduling in other hospitals or can be suitable for scheduling similar to this situation.

## 2. Methods

This section explains the system requirements and the detailed scheme of the proposed system. In the inspection schedule of PET, due to the many conditions and changes to be considered, such as the limitation of the time limit of special drugs, limited medical equipment, and possible rescanning, manual scheduling and time control are quite difficult. This paper proposes a real-time automatic scheduling and control system for PET examination, which can instantly estimate the examination time, allocate medical resources, and respond to possible rescanning in time.

### 2.1. System Requirement

System requirements of the proposed system are described in Definition 1.

**Definition** **1.** *(System requirements). The proposed scheme should meet the following conditions: (1) real-time automatic scheduling: the system performs real-time automatic scheduling according to the patient registration time; (2) scheduling for different medicines: the system schedules the best inspection time according to the cycle time of different medicines; (3) dynamic update of prediction time: when a new patient checks in, or after the end of each examination task of the patient, the system immediately updates the expected prediction task time of each patient; (4) providing predictive inspection time immediately: the system provides the predicted inspection time to the medical staff, patients, and their families in real time; (5) automatic allocation of general and specific beds: the system automatically allocates suitable beds to patients waiting for beds according to all patients’ inspection task completion status; (6) automatic detection of all patients’ tasks periods: the system automatically detects the start time and end time of each examination task of all patients; (7) automatic detection and prediction of examination room conditions: automatic detection and prediction of the use and idle periods of each examination room; (8) instant rescheduling: the system automatically arranges patients who need to be rescanned.*

### 2.2. Details of the Proposed System

**Figure 1:**
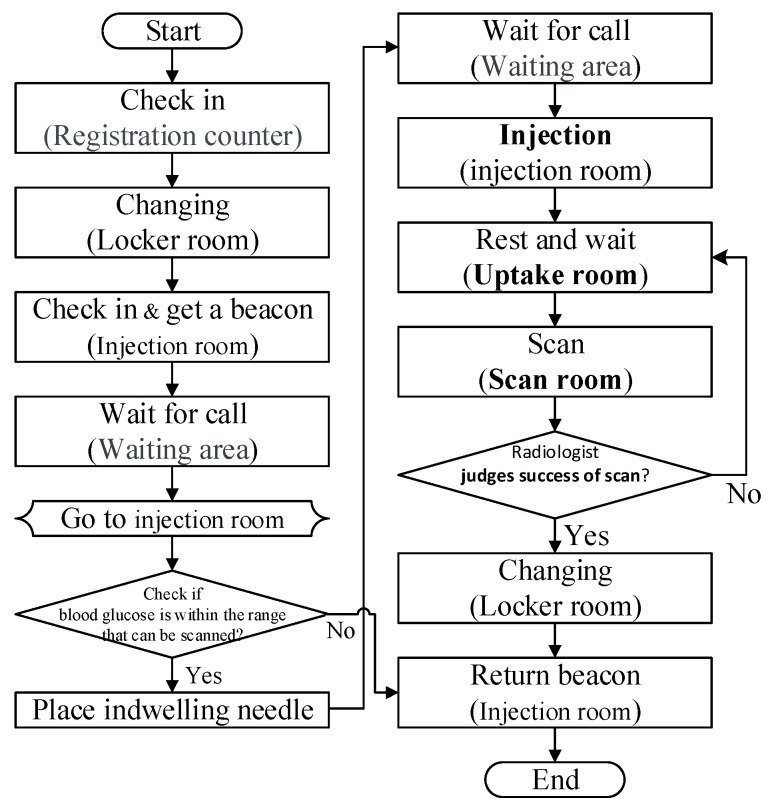
Positron emission tomography (PET) patient examination process.

**Figure 2:**
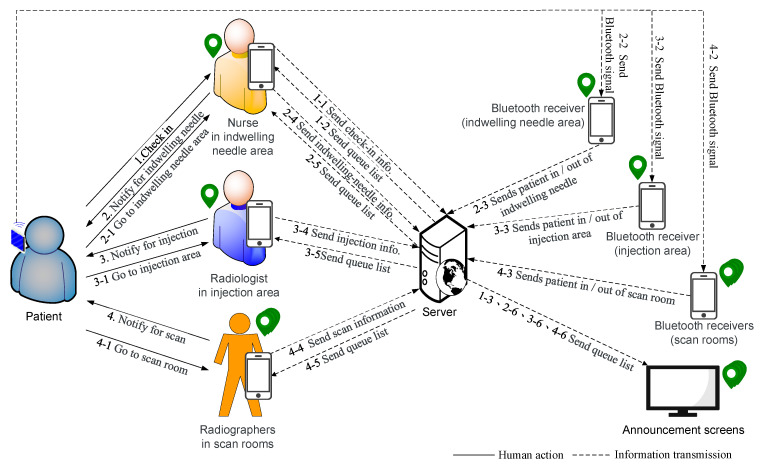
Schematic of automatic scheduling system.

The traditional PET examination process is shown in Figure 1. According to Definition 1 and Figure 1, we propose a new scheduling and control system (Figure 2), which contains five roles and eight phases.

The five roles are patient $P_{i}$, medical staff MS, radiographer $RD_{j}$, server S, and announcement screen, where MS is at the indwelling needle area in the IV indwelling needle room (or called the outer injection room) $R_{IV}$ and at the indwelling needle desk we equipped with a mobile device $D_{MS}$ and a beacon signal receiver $RC_{MS}$. We also equipped with a radiographer $RD_{j}$, a mobile device $D_{RD_{j}}$, and a beacon signal receiver $RC_{j}$ in the *j*-th examination room and on the injection area in the inner injection room. Both $RC_{MS}$ and $RC_{j}$ have functions to detect the content and strength of Beacon signals. Table 1 defines the symbols and parameters used in the proposed method.

The system is divided into seven phases and two algorithms: initial phase, patient check-in phase, patient indwelling needle phase, bed allocation phase, algorithm for scheduling examination room, estimating injection time and scan time, injection phase, scanning phase, end examination phase, and algorithm to determine Beacon’s entry and exit from a certain area.

#### 2.2.1. Initial Phase

S let $A_{IV}=\emptyset$, $A_{inj}=\emptyset$, $A_{BR}=\emptyset$, $A_{R_{k}}=\emptyset$, $T_{ES}(R_{k})$ = 17:00, and the average task times ${\bar{t}}_{IV}(0)$, ${\bar{t}}_{inj}(0)$, and ${\bar{t}}_{ex}(0)$ be their average time of the previous week.

#### 2.2.2. Patient Check-In Phase

**Figure 3:**
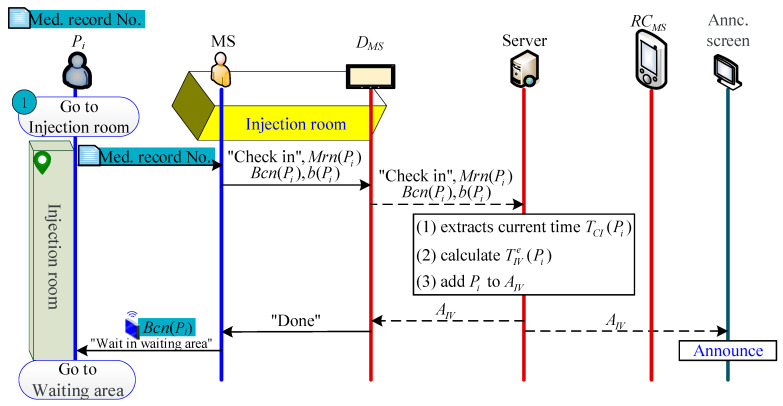
Check-in process.

When $P_{i}$ checks in, MS configures a Beacon for $P_{i}$, uses $D_{MS}$ to scan $P_{i}$’s medical-record-number barcode $Mrn(P_{i})$, enters $P_{i}$’s body status $b(P_{i})$ and Beacon serial number $Bcn_{j}$, and sends $Mrn(P_{i})$, $b(P_{i})$, and $Bcn_{j}$ to S. Then, S calculates the estimated IV indwelling time as follows:

(1) $$T_{IV}^{e}(P_{i})=\{\begin{array}{l} \mathrm{current}\;\mathrm{time},\;\mathrm{if}\;A_{IV}=\emptyset \\ T_{IV}^{e}(P_{i-1})+{\bar{t}}_{IV}(n),\;\mathrm{else} \end{array},$$

followed by adding $Mrn(P_{i})$ to $A_{IV}$. Finally, the patient waits in waiting area. The check-in process is shown in Figure 3.

#### 2.2.3. Patient Indwelling Needle Phase

When the $P_{i}$’s previous patient (i.e., $P_{i-1}$ or $P_{j}$) indwelling needle ends and leaves $R_{IV}$, S will receive the message that $P_{i-1}$ (or $P_{j}$) has left $R_{IV}$, and then automatically transfer $A_{IV}(1)$’s $Bcn(P_{i})$ to $RC_{MS}$, letting $RC_{MS}$ know that the next beacon to be determined whether the signal is to enter or leave is $Bcn(P_{i})$ (please refer to Section 2.2.9 for the detailed determine method). When the time is one minute before $T_{IV}^{e}(P_{i})$, S sends the indwelling needle notification to $D_{MS}$ (or MS checks $D_{MS}$ and judges that $P_{i}$ can be indwelling), and MS goes to the waiting area to notify $P_{i}$ to go to $R_{IV}$ to indwell needle. Otherwise, the patient can actively enter the room after receiving a notification (by sight (for the patient to see that the time is up), hearing (broadcasting the patient’s name), or touching (by making the patient’s pager vibrate through the wireless paging system).

When $P_{i}$ enters $R_{IV}$, $RC_{MS}$ judges that $P_{i}$ has entered $R_{IV}$ indwelling needle and then informs S. *S* then captures the time of entering $R_{IV}$ as the start time of the indwelling needle $T_{IV}^{a}(P_{i})$, adds $P_{i}$ to $A_{BR}$ and deletes it from $A_{IV}$, and calculates the actual and estimated time difference as follows:

(2) $$t_{IV}^{diff}=T_{IV}^{a}(P_{i})-T_{IV}^{e}(P_{i})$$

S then uses $t_{IV}^{diff}$ to update the estimated indwelling time for patients in $A_{IV}$ as follows:

(3) $$T_{IV}^{e}(a_{IV})=T_{IV}^{e}(a_{IV})+t_{IV}^{diff},\forall a_{IV}\in A_{IV}$$

During $P_{i}$ indwelling, MS transmits $t_{abs}(P_{i})$ to S via $D_{MS}$. Next, S allocates an uptake room for patients in $A_{BR}$, calculates the estimated injection time $T_{inj}^{e}(P_{i})$ and the estimated scan time $T_{EE}^{e}(P_{i})$ (for detailed methods, refer to Section 2.2.4 and Section 2.2.5), and uses $T_{EE}^{e}(P_{i})$ to calculate the $T_{inj}^{e}(P_{i})$ back.

After the indwelling needle is completed, $P_{i}$ leaves $R_{IV}$ to the waiting area to wait for the injection. At this time, $RC_{MS}$ automatically determines that $P_{i}$ is leaving $R_{IV}$ and sends a “$P_{i}$ has left $R_{IV}$” message to S. Nest, S extracts the current time $T_{LIV}^{a}(P_{i})$, and calculates the length of the $P_{i}$ indwelling needle as follows:

(4) $$t_{IV}^{}(n)=T_{LIV}^{a}(P_{i})-T_{IV}^{a}(P_{i})$$

Finally, S calculates the average indwelling time from $P_{1}$ to $P_{i}$ as follows:

(5) $${\bar{t}}_{IV}(n)=\frac{\left(n-1){\bar{t}}_{IV}(n-1)+t_{IV}^{}(n\right)}{n}$$

**Figure 4:**
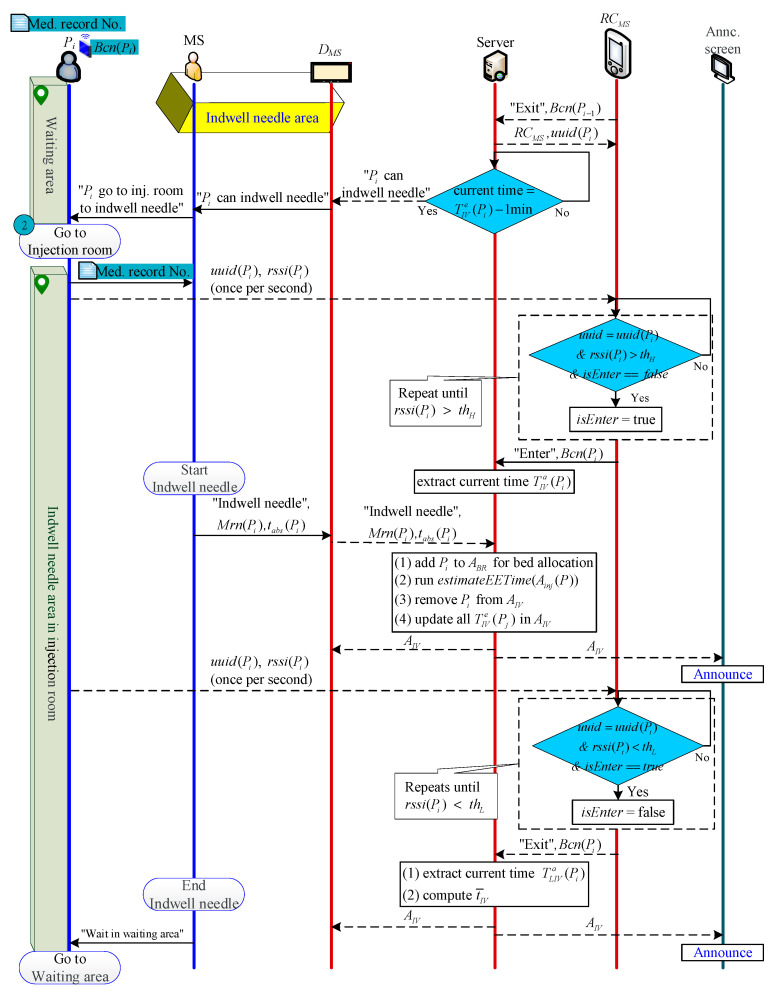
Indwelling needle process.

The indwelling needle process is shown in Figure 4.

#### 2.2.4. Bed Allocation Phase

**Figure 5:**
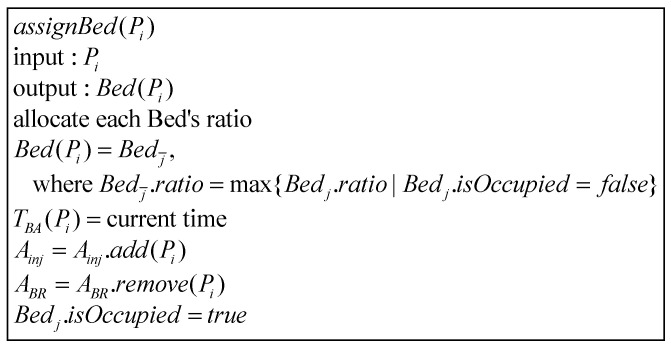
Bed allocation algorithm.

After processing a “successful scan” patient $P_{i}$, $RD_{j}$ uses $D_{RD_{j}}$ to send $T_{IIF}(P_{i})$ to S. S then runs $Bed(P_{i}).isOccupied=false$ to vacate $P_{i}$’s bed, and runs $Bed(P_{j}=A_{BR}(1))=assignBed(P_{j})$ to assign this bed to the next waiting patient $P_{j}$ (Figure 5). Additionally, when the patient $P_{i}$ indwelling needle ends, S performs the bed allocation algorithm $Bed(P_{i})=assignBed(P_{i})$ to try to assign an vacated bed to $P_{i}$.

There are three types of beds in the uptake room, and all three types can be allocated to general patients. However, bedridden patients can only use type-A beds, while patients with short drug absorption times (i.e., $t_{abs}=30$) are often allocated to type-B beds, while type-C Beds can be dispensed to short-absorption-time or general patients. We use “weight allocation” to calculate the priority of bed allocation in the uptake room. The allocation method is shown in Table 2, where 0 means not applicable.

**Figure 6:**
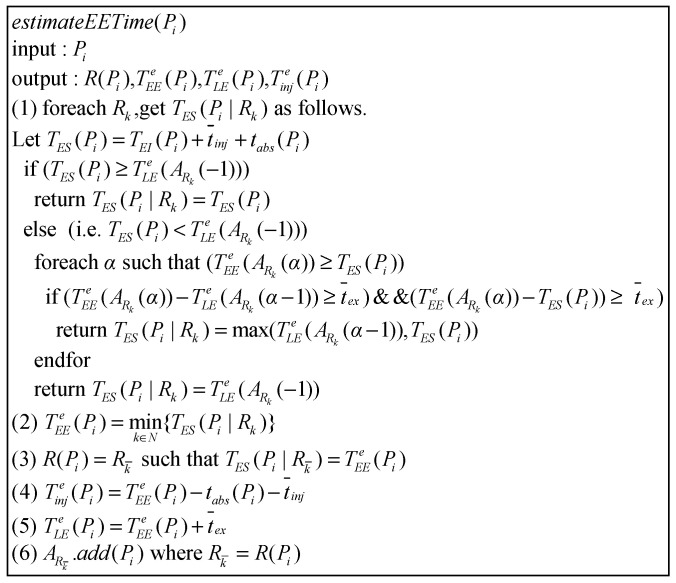
Algorithm for estimating examination process time.

S chooses the unoccupied bed with the highest weight as $Bed(P_{i})$. If $P_{i}$ is allocated to a bed, S extracts the current time as $T_{BA}(P_{i})$, and adds $P_{i}$ to $A_{inj}$ and removes it from $A_{BR}$. S then runs the algorithm for estimating scan time: $estimateEETime(P_{i})$ (Figure 6). Otherwise, if $P_{i}$ is not allocated to any bed, $P_{i}$ continues to wait at $A_{BR}$ until there is a vacated bed in the uptake room.

#### 2.2.5. Algorithm for Scheduling Examination Room, and Estimating Injection and Examination Time

After executing $assignBed(P_{i})$, S then executes the algorithm $estimateEETime(P_{i})$ one by one for patients whose injection has not been estimated in $A_{inj}$, where $estimateEETime(P_{i})$ is used for arranging the examination room $R(P_{i})$, as well as estimating the injection time $T_{inj}^{e}(P_{i})$ and start/end examination time $T_{EE}^{e}(P_{i})$/$T_{LE}^{e}(P_{i})$ (Figure 6). If $P_{i}$ is a patient who has not completed the indwelling needle, S lets $P_{i}$’s fastest injectable time $T_{EI}(P_{i})$ be $T_{BA}(P_{i})$. Otherwise, S lets $T_{EI}(P_{i})$ be $T_{BA}(P_{i})$.

Next, S separately determines which of $T_{ES}(P_{i})$ and $T_{LE}^{e}(A_{R_{k}}(-1))$ is earlier to find out the fastest available scan time $T_{ES}(P_{i}|R_{k})$ in each examination room $R_{k}$, where $T_{LE}^{e}(A_{R_{k}}(-1))$ is the estimated time to leave the examination room $R_{k}$ for the last patient waiting for $R_{k}$. For $R_{k^{\prime }}$, if $T_{ES}(P_{i})\geq T_{LE}^{e}(A_{R_{k^{\prime }}}(-1))$, it means the fastest scan time $T_{ES}(P_{i}|R_{k^{\prime }})$ of $P_{i}$ in $R_{k^{\prime }}$ is $T_{ES}(P_{i})$. Otherwise, it means $P_{i}$ has the opportunity to scan before $T_{LE}^{e}(A_{R_{k^{\prime }}}(-1))$. Therefore, S searches for free time space between $T_{ES}(P_{i})$ and $T_{LE}^{e}(A_{R_{k}}(-1))$.

If there is enough time (higher than the average examination time (${\bar{t}}_{ex}$) in the time space between $T_{LE}^{e}(A_{R_{k}}(\alpha -1))$ and $T_{EE}^{e}(A_{R_{k}}(\alpha ))$ or between $T_{EE}^{e}(A_{R_{k}}(\alpha ))$ and $T_{ES}(P_{i})$, it means $P_{i}$ can perform examinations in the free time gap between “patient $A_{R_{k}}(\alpha -1)$ end scan” and “patient $T_{EE}^{e}(A_{R_{k}}(\alpha ))$ start scan”. Because $T_{ES}(P_{i})$ may fall before $T_{LE}^{e}(A_{R_{k}}(\alpha -1))$, that is, $T_{ES}(P_{i})$ is just when $A_{R_{k}}(\alpha -1)$ is inspecting, this situation is not feasible. Therefore, the fastest time for scan should be $T_{LE}^{e}(A_{R_{k}}(\alpha -1))$ to be a reasonable time. $T_{ES}(P_{i})$ may also fall behind $A_{R_{k}}(\alpha -1)$; hence, the fastest available time can be $T_{ES}(P_{i})$ directly. Therefore, S chooses $T_{ES}(P_{i}|R_{k^{\prime }})$ = max{$T_{ES}(P_{i})$, $T_{LE}^{e}(A_{R_{k}}(\alpha -1))$}. If there is not enough time between $T_{ES}(P_{i})$ and $T_{LE}^{e}(A_{R_{k^{\prime }}}(-1))$, then let $T_{ES}(P_{i}|R_{k^{\prime }})$ be $T_{LE}^{e}(A_{R_{k^{\prime }}}(-1))$.

Finally, S selects the minimum value from $\left\{T_{ES}(P_{i}|R_{j})|\forall j=1,2,\ldots \right\}$ as $T_{EE}^{e}(P_{i})$, uses it to compute the estimated injection time $T_{inj}^{e}(P_{i})$ and estimated end examination time $T_{LE}^{e}(P_{i})$, and adds $P_{i}$ to $A_{R_{k}}$.

#### 2.2.6. Injection Phase

When the $P_{i^{\prime }}$ injection ends and leaves $R_{IJ}$, S will receive the message of “$P_{i^{\prime }}$ is leaving $R_{IJ}$”, and then automatically pass $Bcn(P_{i})$ from $A_{inj}(1)$ to $RC_{0}$ to let $RC_{0}$ know the next beacon determining entry or exit is $Bcn(P_{i})$ (please refer to Section 2.2.9 for the detailed Beacon algorithm). When the time is one minute before $T_{inj}^{e}(P_{i})$, S sends an injection notification to $D_{RD_{0}}$. After receiving the notice, $RD_{0}$ goes to the waiting area and tells $P_{i}$ to go to $R_{IJ}$ to inject.

When $P_{i}$ enters $R_{IJ}$, $RC_{0}$ judges that $Bcn(P_{i})$ enters $R_{IJ}$ for injection and informs S. S then extracts the current time as the injection start time $T_{inj}^{a}(P_{i})$ and removes $P_{i}$ from $A_{inj}$.

When $P_{i}$ leaves $R_{IJ}$ to $Bed(P_{i})$ at the end of the injection, $RC_{0}$ judges that $Bcn(P_{i})$ has left $R_{IJ}$ and notifies S. S extracts the current time as the departure time $T_{Linj}^{a}(P_{i})$, and calculates $P_{i}$’s injection length as follows:

(6) $$t_{inj}^{n}=T_{Linj}^{a}(P_{i})-T_{inj}^{a}(P_{i})$$

Finally, S calculates the average injection time from $P_{1}$ to $P_{i}$ as follows:

(7) $${\bar{t}}_{inj}(n)=\frac{(n-1){\bar{t}}_{inj}(n-1)+t_{inj}^{n}}{n}$$

**Figure 7:**
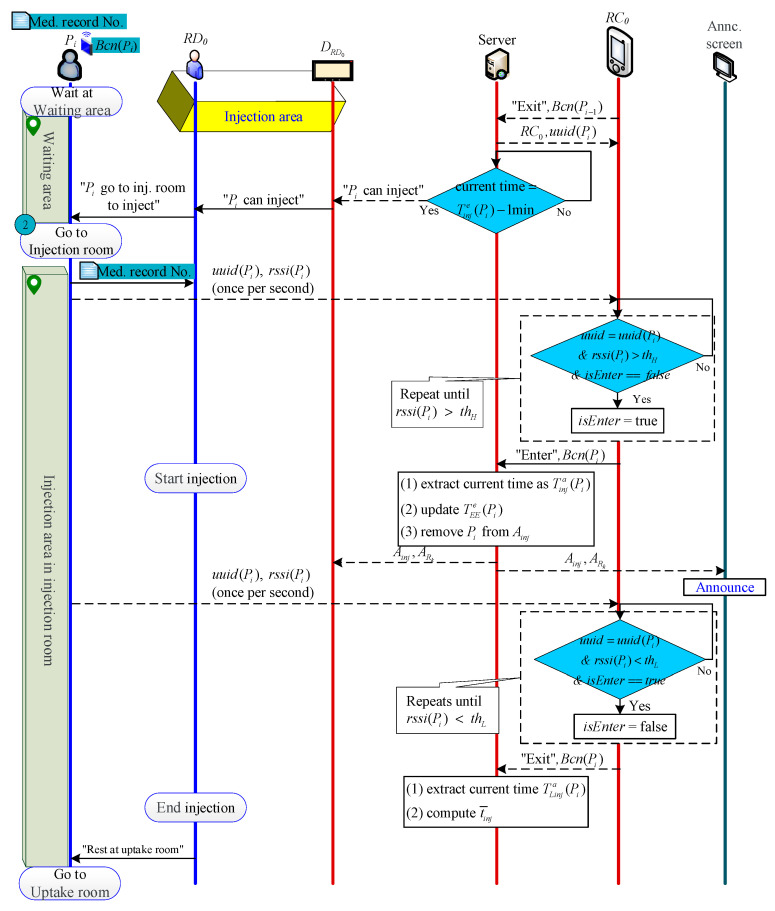
Injection process.

The patient injection process is shown in Figure 7.

#### 2.2.7. Scanning Phase

When the $P_{i^{\prime }}$ examination ends and leaves $R_{k}$, S will receive the message of “$P_{i^{\prime }}$ is leaving $R_{k}$”, and then automatically pass $Bcn(P_{i})$ from $A_{R_{k}}(1)$ to $RC_{j}$ to let $RC_{j}$ know the next beacon determining entry or exit is $Bcn(P_{i})$ (please refer to Section 2.2.9 for the detailed Beacon algorithm). When the time is 5 min before $T_{EE}^{e}(P_{i})$, S sends an examination notification to $D_{RD_{j}}$. After receiving the notice (or $RD_{j}$ determines that $P_{i}$ can be scanned via checking $D_{RD_{j}}$), $RD_{j}$ goes to the uptake room and tells $P_{i}$ to go to $R_{k}$ to wait for examination.

When $P_{i}$ enters $R_{k}$, $RC_{j}$ judges that $Bcn(P_{i})$ enters $R_{k}$ and informs S. S then extracts the current time as the examination start time $T_{EE}^{a}(P_{i})$ and removes $P_{i}$ from $A_{R_{k}}$.

If $RD_{j}$, which monitors the patient’s photographic image in the background, judges that $P_{i}$’s scan image is successful, they use $D_{RD_{j}}$ to transmit $T_{IIF}(P_{i})$ to S. S empties $Bed(P_{i})$, and then automatically allocates the bed to a patient in $A_{BR}$. In addition, $P_{i}$ needs go to $R_{IV}$ to pull the needle and return the Beacon to complete the inspection.

If $RD_{j}$ judges that the scan image of $P_{i}$ fails, then $P_{i}$ needs to be scanned again. $RD_{j}$ uses $D_{RD_{j}}$ to select a patient so that $P_{i}$ should be rescanned after the patient’s end examination. S then updates all the estimated examination times of the patients who are injected in $A_{R_{k}}$, clears the estimated injection and examination times of all patients in $A_{inj}$, and re-executes $estimateEETime(P_{i})$ to calculate the new estimated injection and examination times of all patients.

When $P_{i}$ leaves $R(P_{i})$ at the end of examination, $RC_{j}$ judges that $Bcn(P_{i})$ has left $R(P_{i})$ and notifies S. S extracts the current time as the departure time $T_{LE}^{a}(P_{i})$, and calculates $P_{i}$’s examination length as follows:

(8) $$t_{ex}^{n}=T_{LE}^{a}(P_{i})-T_{EE}^{a}(P_{i})$$

Finally, S calculates the average scan time from $P_{1}$ to $P_{i}$ as follows:

(9) $${\bar{t}}_{ex}(n)=\frac{(n-1){\bar{t}}_{ex}(n-1)+t_{ex}^{n}}{n}$$

**Figure 8:**
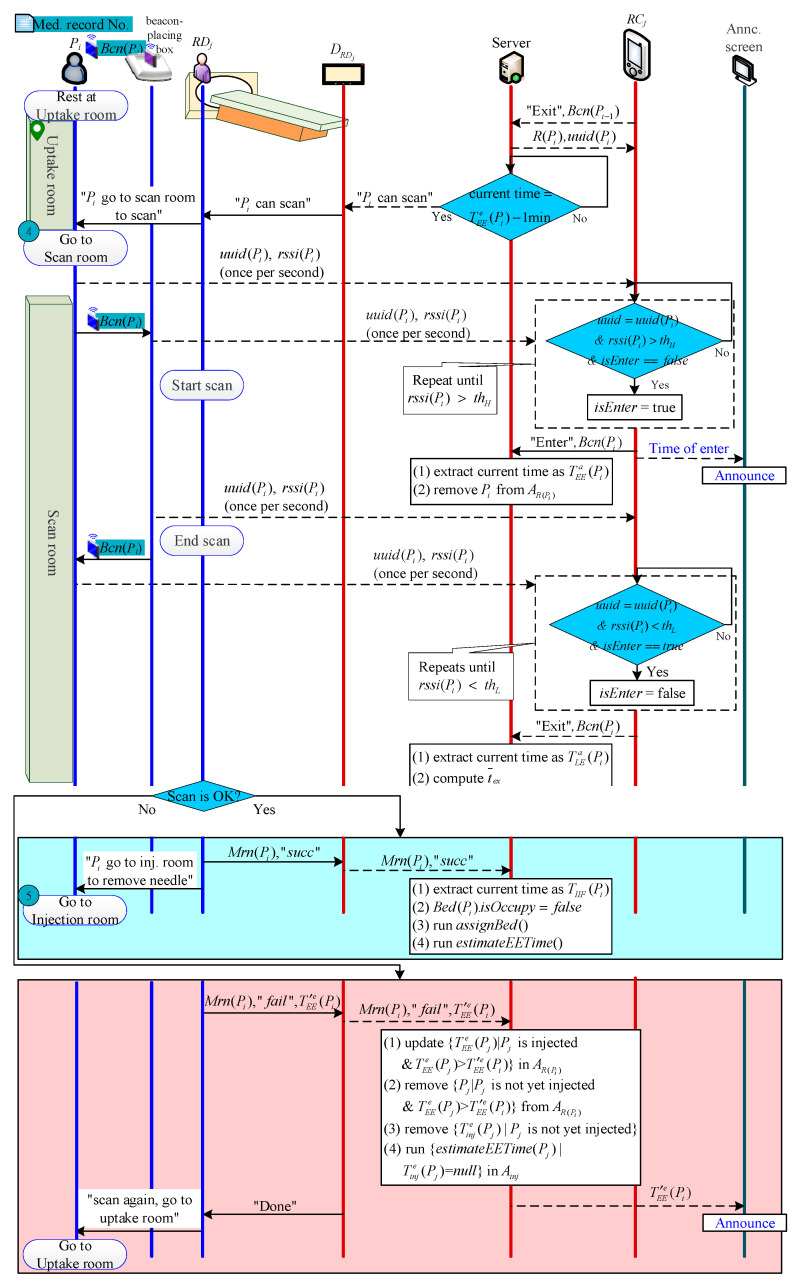
Examination process.

The patient examination process is shown in Figure 8.

#### 2.2.8. End Examination Phase

**Figure 9:**
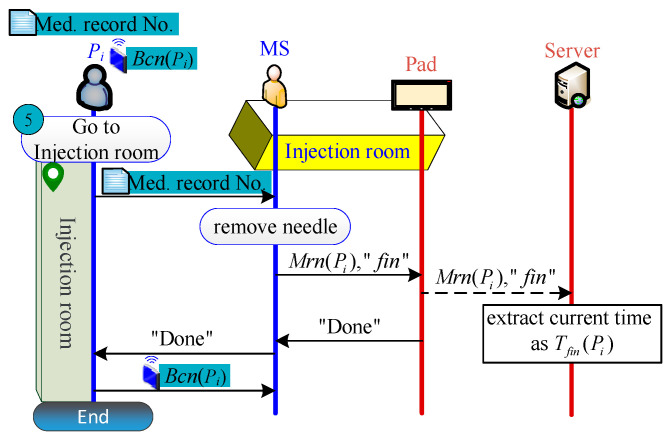
End examination process.

Patient $P_{i}$, who was successfully examined, would return to the injection room, remove the needle, and return their Beacon. MS then clicks the end button via $D_{MS}$ to inform S, and completes the PET inspection (Figure 9).

#### 2.2.9. Algorithm to Determine Beacon’s Entering or Leaving from a Certain Area

**Figure 10:**
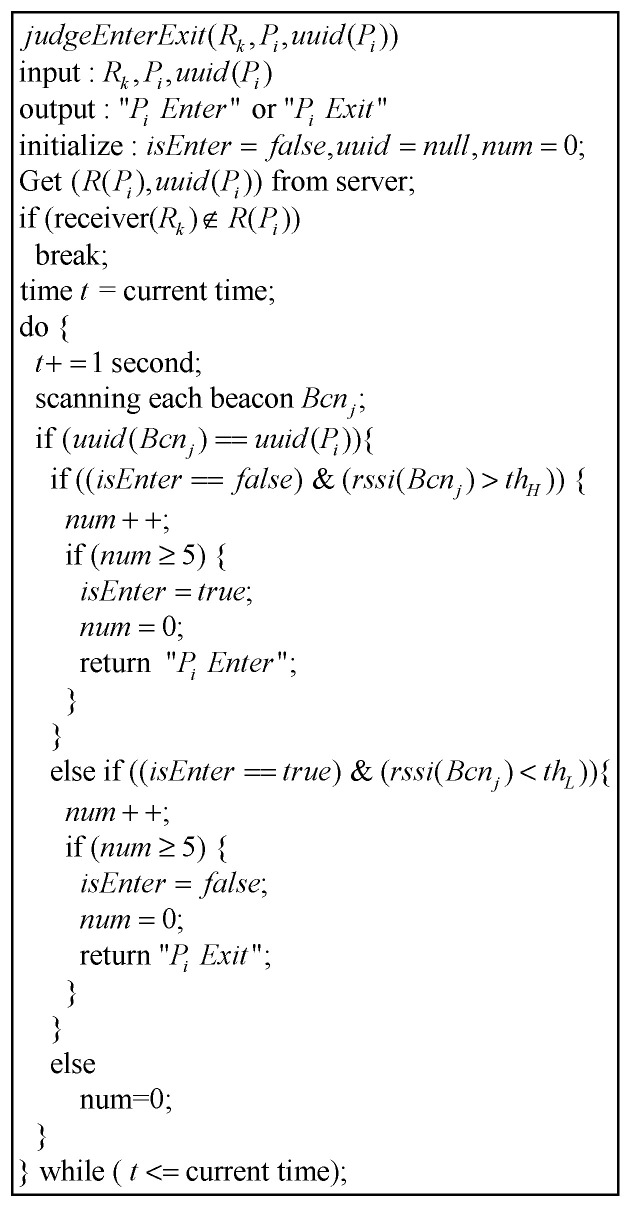
Algorithm to determine Beacon’s entering/leaving from a certain area.

$RC_{MS}$ or $RC_{j}$ can judge whether $Bcn(P_{i})$ enters/leaves an area on the basis of whether $rssi(Bcn(P_{i}))$ is “greater than $th_{H}$” or “less than $th_{L}$” (Figure 10). However, when $P_{i}$ approaches the entrance to the area, the value of $rssi(Bcn(P_{i}))$ will oscillate back and forth between $th_{H}$ and $th_{L}$, making it impossible to determine whether $P_{i}$ enters or leaves the area. Therefore, we designate that $rssi(Bcn(P_{i}))$ must be detected greater than $th_{H}$ (or less than $th_{L}$) more than five consecutive times before it can be judged as entering (or leaving).

## 3. Materials

We first collected the status and time data of various tasks and examinations of 200 actual patients at the Linkou Chang Gung Hospital as a PET medical database and control group. We also implemented the proposed system in the Android system, and we fed the patient’s physical condition, check-in time, and rescan status of the PET medical database data collected above into our implementation system, using the obtained time data as the experimental group. Lastly, we carried out data analysis to compare and analyze the results of the experimental group and the control group.

### 3.1. Data Collection of Control Group

**Figure 11:**
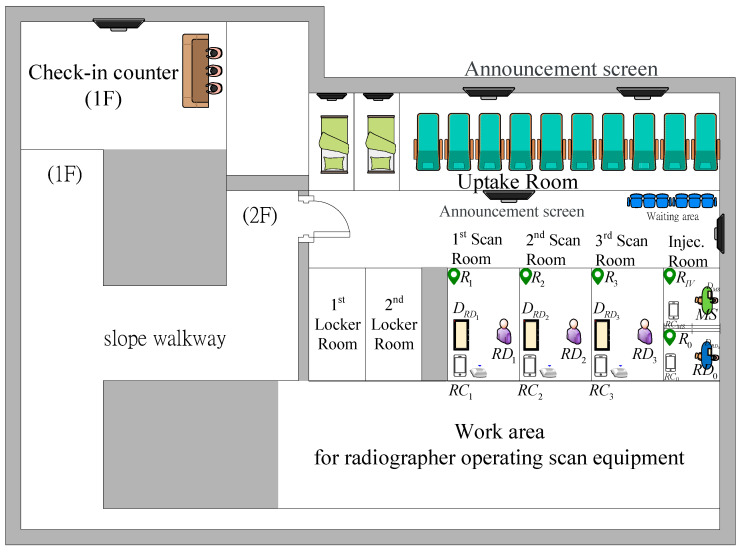
PET outpatient area layout of Linkou Chang Gung Hospital.

We set up receivers in five places in Linkou Chang Gung Hospital (Figure 11). We let actual patients wear the Beacon and perform PET inspection procedures under the current manual scheduling method, and we recorded time data and examination information of 200 patients for two consecutive weeks (12 days).

We used a desktop computer (using the Windows 10 operating system, Intel (R) Xeon (R) central processing unit (CPU) E3-1230 with a 3.3 GHz and 3.7 GHz processor and 8 GB random-access memory (RAM) as the server to collect data, calculate, and process communication matters. We also used four Android 5.0 operating system phones, including three HTC Desire816 (Qualcomm S400 1.6 GHz quad-core processors) as Beacon signal receivers in three scan rooms to determine patients’ location in or out of the room, while another HTC One E8 (Qualcomm S801 2.5 GHz quad-core processor) was responsible for inputting and transmitting patient examination information to the server. We chose THLight’s B3029 T as the Beacon for each patient.

As for the software, we used Android Studio and JAVA to develop our own programs, adopted the library provided by THLight for Beacon control and signal reading, and chose MySQL database tools as the back-end database collection.

The system program structure of data collection was divided into six parts according to different tasks: nurse device during check-in phase, nurse device during indwelling needle, radiographer device during injection, radiographer app on the scan room side, server, and receivers in scan rooms. The communication method between the server and the mobile devices of the medical staff was Wi-Fi. The screens of the mobile phone app operated by the medical staff at each inspection stage are described below.

#### 3.1.1. Patient Check-In

**Figure 12:**
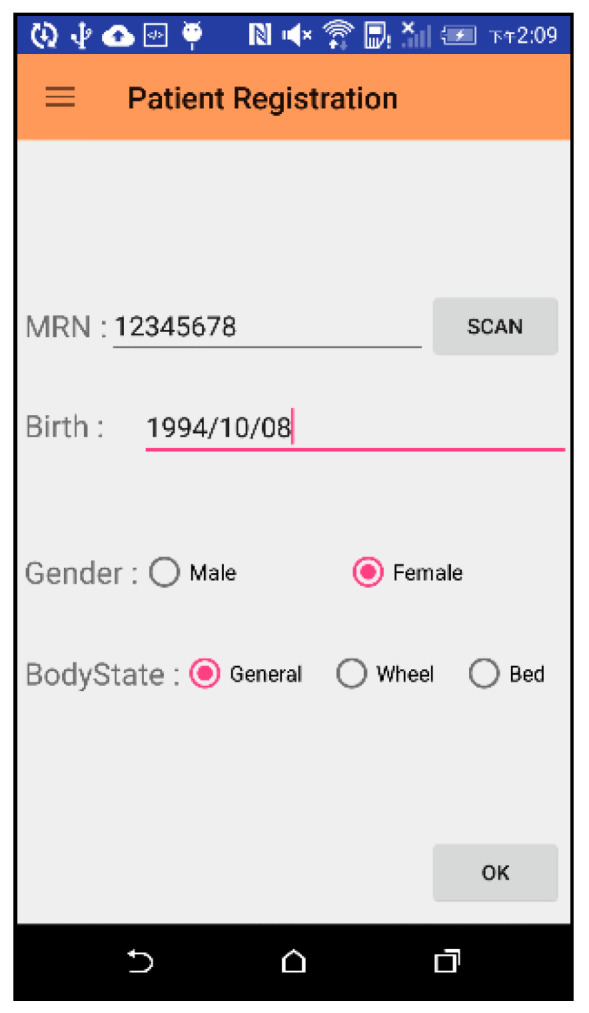
Operation screen of patient check-in phase.

When the patient checks in, the nurse at the needle indwelling area of the injection room enters the patients’ report information screen. Because there are many numbers in the patients’ medical record number, it is easy to make mistakes when manually inputting it. Therefore, the scan button “SCAN” was designed to scan the one-dimensional barcode of the medical record number to reduce error (Figure 12).

#### 3.1.2. Indwelling Needle and Injection Phase

**Figure 13:**
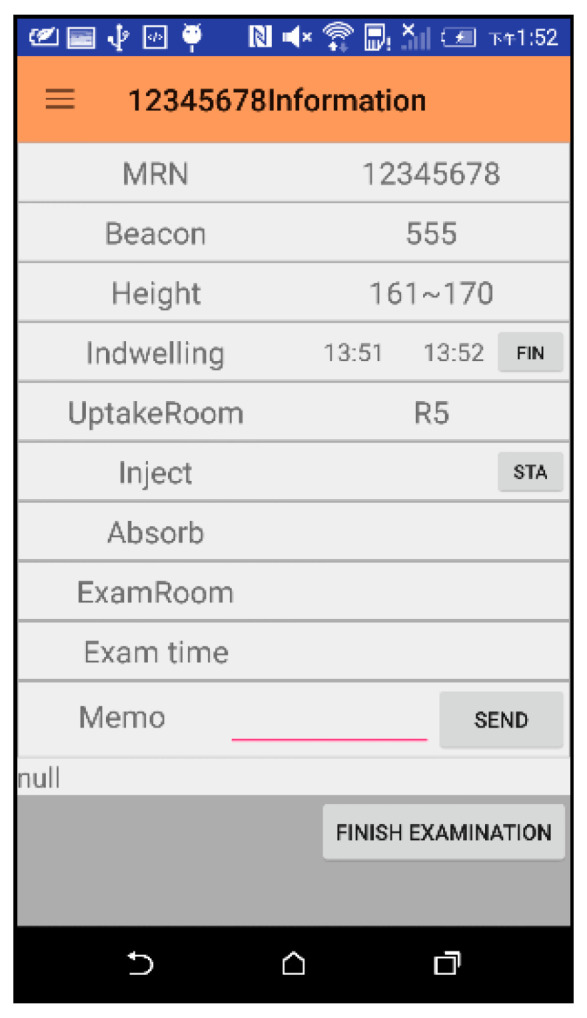
Interface for indwelling needle and injection phase.

The interfaces of the medical staff during the indwelling phase and during the injection phase are the same (Figure 13). The medical staff enters the patient information required for the examination. When the needle or injection is started, the start button “STA” is clicked to capture the start time. When the indwelling needle or injection ends, the button is clicked again to grab the end time.

#### 3.1.3. Patients’ Queue List and Scan Room Waiting List for Each Scan Room

**Figure 14:**
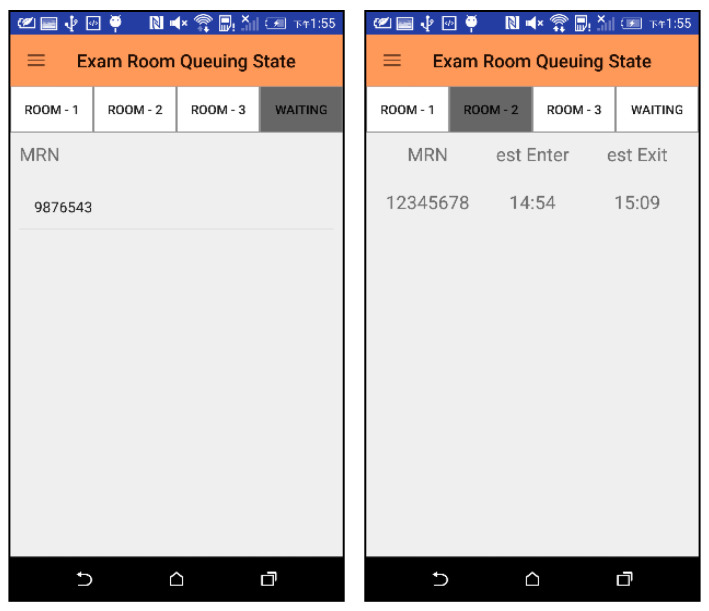
Waiting list for scan rooms and patient queues for each scan room.

The information of patients who have completed the indwelling needle but have not yet arranged a scan room is shown on the left in Figure 14 for the radiographer *RD*_0_ at the injection phase to view and arrange the scan room. The radiographer can click on the MRN number (i.e., “9876543”) to go to the interface of Figure 13 to enter scan information. For patients who have been assigned a scan room, their information is displayed on each page according to each different scan room (shown on the right of Figure 14).

#### 3.1.4. Total Queue List

**Figure 15:**
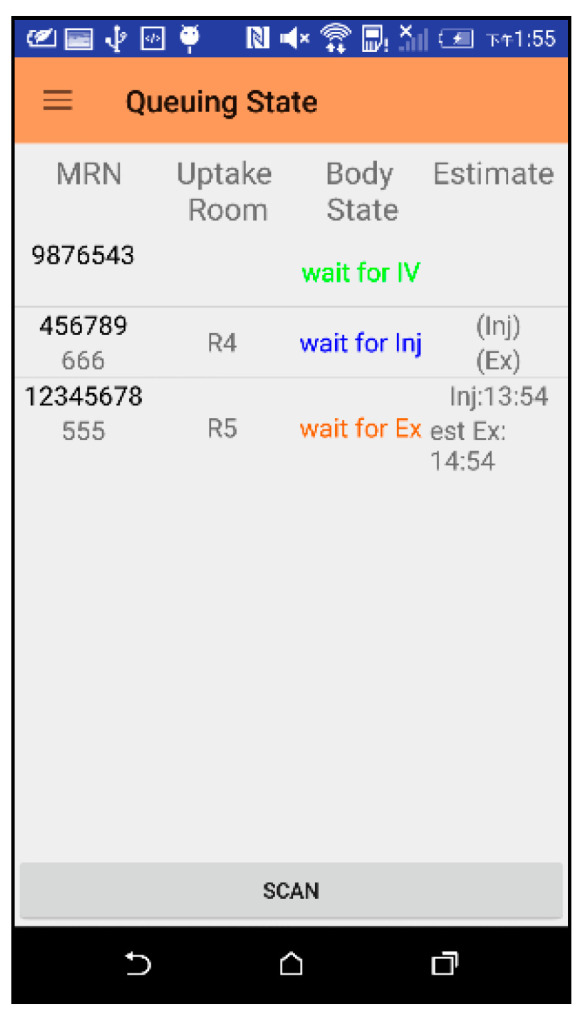
Total queue list interface.

Patients who have already checked in and have not completed the examination will be displayed in the screen of Figure 15 for medical staff at each stage of the examination. Clicking on the MRN number will jump to the interface of Figure 13 for medical staff to enter or view detailed information. When there are a large number of patients, it is not easy to find the medical record number to view detailed information. We can use the “SCAN” button to scan the MRN barcode of a patient, and the system will directly display the detailed information of the patient.

### 3.2. Data Collection of Experimental Group

**Figure 16:**
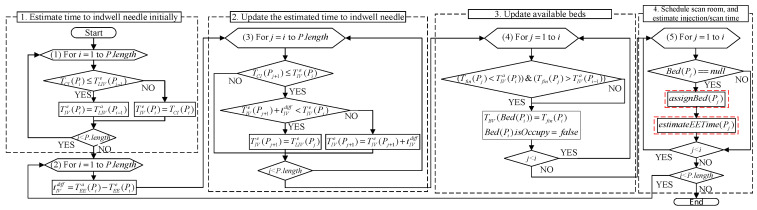
Experimental group data output flow chart.

We then fed these 200 PET medical database data on the patients’ physical condition, report time, and rescan status into our proposed system for the output of experimental group data (Figure 16).

#### 3.2.1. Estimate Time to Indwell Time Initially

Initially, the program downloads one day’s data from the database, and then calculates the initial estimated indwelling time for all patients.

#### 3.2.2. Update the Estimated Time to Indwell Needle

After the initial estimated indwelling needles for all patients have been calculated, the patients are sequentially entered into the next phase of the loop, i.e., to update the estimated time to indwell needle. The value of *i* in the first loop indicates the serial number of the patient who started the indwelling needle, and the patient is represented by $P_{i}$. The program subsequently uses the time of $P_{i}$’s indwelling needle as the current time, and then uses this time to determine the sequence of other times. The program first calculates the difference $t_{IV}^{diff}$ between the actual and estimated indwelling time of $P_{i}$. After calculating the content via executing the second loop, the program will update the estimated indwelling time for all patients after $P_{i}$. Because not every patient checks in before the $P_{i}$’s indwelling needle, it is necessary to judge the timing of $T_{CI}(P_{j+1})$ and $T_{IV}^{a}(P_{i})$ first. If the patient $P_{j}$ has already checked in before $T_{IV}^{a}(P_{i})$, the program will update $T_{IV}^{e}(P_{j})$ for the patient. The detailed algorithm is shown as Equation (1) in Section 2.

#### 3.2.3. Update Available Beds

After updating the estimated indwelling time, the program then updates the available beds. The first loop searches for all patients who have been scanned before the $P_{i}$’s indwelling needle and determines whether there is a $T_{IIF}(P_{j})$ between $T_{IV}^{a}(P_{i})$ and $T_{IV}^{a}(P_{i-1})$. If there is, it means a patient’s bed can be vacated during this time gap, with a bed vacancy at $T_{IIF}(P_{j})$.

#### 3.2.4. Schedule Scan Room and Estimate Injection/Scan Time

After updating the available beds, the program executes the fourth procedure to allocate beds for patients $P_{j}$ who have not been allocated a bed before $T_{IV}^{a}(P_{i})$. If $P_{j}$ is assigned to a bed, the program then proceeds with their estimated time of scan and injection.

A. Arrange beds

**Figure 17:**
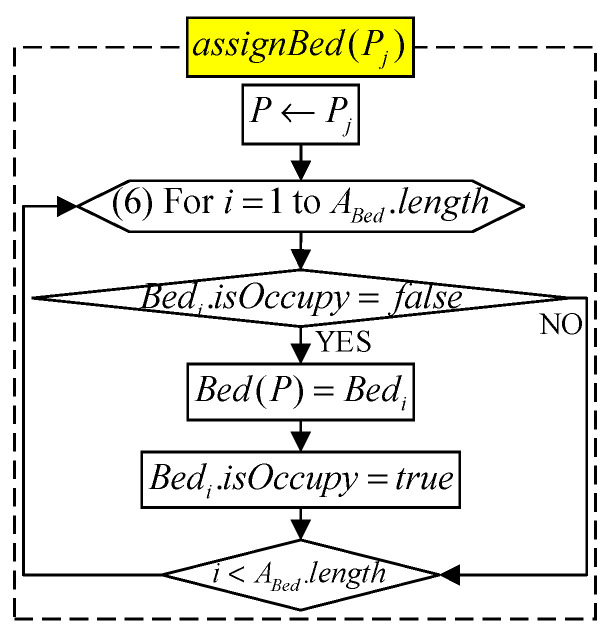
Flow chart for arranging beds.

The bed allocation algorithm $assignBed(P_{j})$ is shown in Figure 5 of Section 2, and the program flow is shown in Figure 17.

B. Estimate injection time and scan time

**Figure 18:**
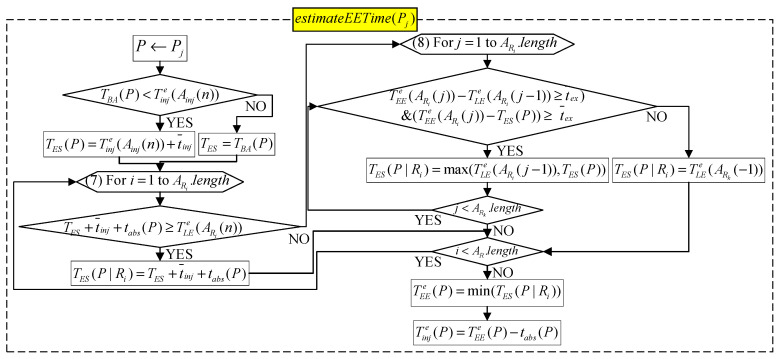
Flow chart for estimating injection time and scan time.

For patient $P_{j}$ assigned to a bed, the program then performs $estimateEETime(P_{i})$ to estimate the injection and scan time. The algorithm is shown in Figure 6 of Section 2, and the program flow is shown in Figure 18.

## 4. Discussion

In this section, we compare and analyze the experimental group and the control group, and we discuss the results in four parts.

We first calculated the average time difference between the two groups at three start times (i.e., indwelling needle, injection, and scan) for 12 days to see if there was an error between the time calculated by the experimental group and the actual time of the control group (Table 3.) “Absolute value” and “relative value” respectively represent “with” and “without” positive and negative values, i.e., the average value of the “absolute value” and “relative value” of each experimental group time minus the control group time on a certain day. We also explored whether the estimated time by using the proposed automatic scheduling method can be faster than the original manual scheduling time, and what the proportion of time earlier is (that is, how many patients would benefit from this). The “ahead rate” represents the proportion where the estimated times of the experimental group was earlier than the actual times of the control group.

We then analyze and discuss the proposed method in four parts.

### 4.1. Analysis of Indwelling Needle Time

The last update time for each patient was the time the previous patient entered the scan room. Comparing the final data with the actual data collected, the average time difference was about 2.94 min (Table 3). The maximum value (time early up) and minimum value (time late up) were 17 and −12 min, respectively (Table 4).

### 4.2. Analysis of Injection and Scan Times

At the stage of estimating the injection, the time difference may be caused by the pharmacy cycle time having a buffer time of ±10 min and the radiographer manually calculating the estimated time. According to our observations at the scene, the radiographer only grasped the approximate time during the manual calculation, resulting in extra time between the end of the injection by the previous patient and the time when the next patient started the injection. In addition, when the indwelling needle ends on each occasion, the nurse must call the pharmacy to ask the pharmacist to give the medicine, and the radiographer must wait for the pharmacist to give the medicine before injecting the patient. These actions take up the time of the patient inspection process. However, the method proposed in this article can remove the above problems. If the medical staff performs the inspection process according to the estimated time calculated by the app, the injection process can be advanced 6.14 min on average (Table 3).

During the estimated scanning stage, the radiographer tries to let the patient scan 5–10 min in advance. However, the proposed algorithm was designed according to the standard pharmaceutical cycle time, allowing the radiographer to adjust the scan according to the standard time. This resulted in a high time difference (Table 4) and low ahead rate (Table 3).

Furthermore, the reason for the standard deviation being larger during the injection phase and the photographic phase than during the indwelling needle phase is that some machines were idle for less than the average imaging time of the patient, but some radiographers still arranged the patient in the gap to reduce the idle time of the imaging equipment. In addition to causing a time difference, this also made it very compact for patients who needed to be scanned again in the future.

In addition to the abovementioned causes of time differences in injection and scan phases, unexpected situations can sometimes occur on the scene, and the delay of the process is also one of the reasons for the time differences.

### 4.3. Analysis of Allocating Beds

Because bedridden patients require larger space, some require special equipment, and the types of uptake rooms vary, if the general patients occupy the uptake room of the bedridden patient, this can lead to prolonged waiting time of the bedridden patient. The proposed weight allocation method (Table 2) (i.e., using *b*() function) could shorten the average waiting time from indwelling needle to injection by 3.29 min (Table 5), save 40.35% of patient waiting time, and effectively solve this problem.

### 4.4. Overall Improvement

After analysis, the proportion of patients with a time difference of less than 10 min reached 68%, indicating that about 70% of the values were close to the actual situation, achieving high accuracy. In addition, 82% of the patients had an average reduction of 6.14 min (according to the estimated injection time), which verified the high efficiency of the proposed system.

## 5. Comparison

This section analyzes and compares the properties including the eight requirements in Definition 1 and other features. Table 6 displays the comparisons for the proposed automatic scheduling system and the current manual scheduling system, and Table 7 summarizes the comparison of the properties for the proposed system and those schemes proposed by Xiao et al. [5], Tantitharanukul and Throngjai [9], and Luscombe and Kozan [12].

### 5.1. Comparison with Proposed and Current Scheduling System

We functionally compared the current manual system with the proposed automatic system (Table 6). Our system has four automatic functions to solve the problem of smooth processes. We also compared the two systems in terms of “the time required” for medical staff or patients to process each task. The details are given below.

- Scheduling of patient examination order: The system automatically orders the order of patient examinations, eliminating the need for radiographers to manually order.
- Scheduling of each inspection time: The system calculates the best patient examination time for different drug cycle times, and the radiographer does not need to calculate it manually.
- Patient interrupts MS workflow by asking questions about examination time: The system calculates the estimated time of each inspection task and announces it to medical staff and patients, reducing the number of times when medical staff are interrupted by patient inquiries.
- Dispensing reminder: The pharmacist checks the app’s patient injection status to dispense medicine, eliminating the need for MS to call and let the pharmacist dispense medicine.
- Patient estimated time reminder: The system sends a reminder message when the estimated time for each patient task is approaching.
- Patient and family inquiries: Patients and family members can check the examination time by themselves, reducing the number of inquiries.

### 5.2. Comparison of Proposed and Previous Works

According to the system requirements proposed in Definition 1, we compared our method with previous works (Table 7). The first five functions could only be totally achieved by our method, and the sixth, seventh, and eighth functions could only be provided by our system. In the PET patient scheduling problems, the biggest issue with the scan room and nurse manual scheduling is that the patient’s examination fails and these examinations need to be repeated. Therefore, immediate rescheduling is the most important function, and only our method can completely solve this problem.

## 6. Conclusions

Due to the limited equipment resources and cycle time of PET, it is not an easy task for medical staff to calculate time and allocate resources on their own. This paper proposes a method for real-time estimation and scheduling of multi-resource allocation and pharmacy limitation, combined with Beacon to automatically detect patient entry and exit, and effectively control the inspection process. The results showed that the time difference between the experimental group and the control group was about 2.94 min, 7.95 min, and 7.32 min in the indwelling needle phase, the injection phase, and the scanning phase, respectively. Moreover, the proportion of patients with a time difference of less than 10 min reached 68%, indicating that most values were close to the actual situation, achieving high accuracy. In addition, 82% of the patients had an average reduction of 6.14 min, verifying the high efficiency of the proposed system. Therefore, the proposed system not only reduces the work of medical staff, but also provides patients and their families with credible estimates of time.

Future research will focus on more detailed data analysis to improve the accuracy of estimated time. In addition, some patients not only have a PET examination but other radiological procedures to be performed on the same day. The radiographer must consider these conditions together to schedule appointments. Future studies can consider combining the scheduling of other radiology departments to make the entire radiological diagnosis process easier and smoother.

## Figures and Tables

**Table 1:** Notations.

Notation	Definition
$P_{i}$	The *i*-th patient
MS	The medical staff (nurse) in $R_{IV}$
$RD_{j}$	The radiographer in the *j*-th scan room
S	Server
$R_{IV}$	IV catheter indwelling room
$R_{IJ}$	Inner injection room
$R_{k}$	The *k*-th examination room
$Bed_{j}$	The *j*-th bed (j = 1, 2, 3, …)
$Beacon_{j}$	The *j*-th Beacon
$rssi_{j}$	Signal strength of $Beacon_{j}$
$th_{H}$/$th_{L}$	Threshold of RSSI strong/weak signals
$D_{MS}$/$D_{RD_{j}}$	Device of MS/$RD_{j}$
$RC_{MS}$/$RC_{j}$	Receivers of MS/$RD_{j}$
$A_{IV}$	List of patients waiting for IV catheter indwelling
$A_{inj}$	List of patients waiting for injection
$A_{BR}$	List of patients requesting beds
$A_{BV}$	List of available beds
$A_{R_{k}}$	List of patients waiting for $R_{k}$
A(α)	The *α*-th element of the list A
A(−α)	The last *α*-th element of the list A
$b(P_{i})$	Body status of $P_{i}$ (bedridden or non-bedridden)
$t_{abs}(P_{i})$	Drug absorption time of $P_{i}$ (e.g., 30 min)
$t_{IV}^{diff}$	Time difference between estimated and actual IV catheter indwelling
$t_{inj}^{diff}$	Time difference between estimated and actual injection
${\bar{t}}_{IV}(\alpha )$	The average IV catheter indwelling time until the *α*-th time
${\bar{t}}_{inj}(\alpha )$	The average injection time until the *α*-th time
${\bar{t}}_{ex}(\alpha )$	The average examination time until the *α*-th time
$Bed(P_{i})$	The bed of $P_{i}$
$Bcn(P_{i})$	The Beacon of $P_{i}$
$uuid(P_{i})$	The UUID of $P_{i}$
$Mrn(P_{i})$	The medical record number for $P_{i}$
$R(P_{i})$	The scan/examination room for $P_{i}$
$T_{CO}(P_{i})$	The examination check-out time for $P_{i}$
$T_{IV}^{e}(P_{i})$/$T_{IV}^{a}(P_{i})$	The estimated/actual IV catheter indwelling time for $P_{i}$
$T_{LIV}^{e}(P_{i})$/$T_{LIV}^{a}(P_{i})$	$P_{i}$’s estimated/actual time to leave the IV room
$T_{inj}^{e}(P_{i})$/$T_{inj}^{a}(P_{i})$	The estimated/actual injection time for $P_{i}$ (e.g., 10:00 a.m.)
$T_{Linj}^{e}(P_{i})$/$T_{Linj}^{a}(P_{i})$	$P_{i}$’s estimated/actual time to leave the injection room
$T_{EE}^{e}(P_{i})/T_{EE}^{a}(P_{i})$	The estimated/actual time to enter the examination room for $P_{i}$ (e.g., 10:00 a.m.)
$T_{LE}^{e}(P_{i})$/$T_{LE}^{a}(P_{i})$	The estimated/actual time to leave the examination room for $P_{i}$ (e.g., 10:00 a.m.)
$T_{BV}(Bed_{j})$	The bed vacated time of $Bed_{j}$
$T_{BA}(P_{i})$	The bed available time for $P_{i}$
$T_{EI}(P_{i})$	The earliest injection time for $P_{i}$
$T_{ES}(P_{i})$	The earliest scan/examination time for $P_{i}$
$T_{ES}(P_{i}|R_{k})$	The earliest scan/examination time at $R_{k}$ for $P_{i}$
$T_{IIF}(P_{i})$	Time for doctor to finish $P_{i}$’s DICOM image interpretation

**Table 2:** Weight allocation in the intake room.

	Bed Type	A	B	**C**
$b(P_{i})$				
**Bedridden**		6	0	0
**General and** $t_{abs}(P_{i})=30$		1	3	2
**General**		1	2	3

**Table 3:** Time difference between the two groups and ahead rate (unit: min).

Time Difference	Indwelling Needle			Injection			Scan		
	Absolute Value	Relative Value	Ahead Rate	Absolute Value	Relative Value	Ahead Rate	Absolute Value	Relative Value	Ahead Rate
Day 1	4.17	−1.00	33%	4.91	−4.00	91%	7.00	0.25	18.2%
Day 2	3.62	−1.32	58%	6.50	−6.50	100%	5.33	−4.17	75%
Day 3	1.67	−0.92	33%	12.36	−12.36	100%	5.91	−0.43	64%
Day 4	4.73	−0.58	2%	4.20	−4.00	80%	9.20	1.20	30%
Day 5	1.67	−1.00	54%	14.91	−14.91	100%	8.73	−0.53	64%
Day 6	2.93	−1.32	53%	8.21	−8.21	100%	5.71	1.00	35.7%
Day 7	2.38	−0.92	50%	11.27	2.87	93%	12.33	1.01	13%
Day 8	1.88	−0.58	31%	5.20	−4.93	53%	3.93	2.33	33%
Day 9	2.71	−1.00	71%	5.77	−0.85	54%	7.85	0.78	0%
Day 10	2.79	−1.32	63%	7.31	−6.69	94%	7.50	−3.25	56%
Day 11	3.25	−0.92	62%	4.65	−4.48	78%	5.74	0.96	43%
Day 12	3.53	−0.58	57%	10.06	−9.61	89%	8.56	−6.11	72%
Average	2.94	−0.96	47%	7.95	−6.14	86%	7.32	−0.58	42%
Standard error	0.93	0.26	0.18	3.32	4.61	0.16	2.14	2.45	0.23

**Table 4:** Maximum and minimum time difference between the two groups (unit: min).

	Indwelling Needle	Injection	Scan
Maximum	17	23	33
Minimum	−12	−29	−25

**Table 5:** Average waiting time from indwelling needle to injection.

	Without *b*()	With *b*()
Day 1	6 min	2 min
Day 2	18 min	16 min
Day 3	3 min	1 min
Day 4	10 min	6 min
Day 5	14 min	7 min
Day 6	1 min	1 min
Day 7	5 min	1 min
Average	8.14 min	4.86 min

**Table 6:** Comparison of proposed and current scheduling systems (unit: s).

	Tasks	Current System	Proposed System
Medical staff	A. Order scheduling	Manual scheduling (10 s)	App automatic scheduling (0 s)
	B. Inspection time scheduling	Manual calculation (60 s)	App automatic calculation (1 s)
	C. Being interrupted	MS was interrupted due to patients inquiry times (30 s)	MS was not interrupted because patients watched the screen by themselves (0 s)
	D. Dispensing reminder	MS calls pharmacist (30 s)	Pharmacist checks the APP device (0 s)
	E. Patient estimated time reminder	MS pay attention to patient examination time (anytime)	MS waits for APP notification (0 s)
Patient (family)	F. Time inquiry	Manual inquiry MS (30 s)	View announcements (5 s)

**Table 7:** Comparison of proposed and previous works.

	[5]	[9]	[12]	Proposed
(1) Real-time automatic scheduling			v	v
(2) Scheduling for different medicines	v			v
(3) Dynamic update of prediction time		v	v	v
(4) Immediate provision of predictive inspection time		v		v
(5) Automatic allocation of general and specific beds			v	v
(6) Automatic detection of all patients’ tasks periods				v
(7) Automatic detection and prediction of examination room conditions				v
(8) Instant rescheduling				v

## Data Availability

The statistical data presented in this study are available in Table 3, Table 4 and Table 5. The datasets used and/or analyzed during the current study are available from the corresponding author upon request.
